# In Silico Investigation of TATA-Binding Protein as a Therapeutic Target for Chagas Disease: Insights into FDA Drug Repositioning

**DOI:** 10.3390/ph18060845

**Published:** 2025-06-04

**Authors:** Carlos Gaona-López, Domingo Méndez-Álvarez, Alonzo Gonzalez-Gonzalez, Guadalupe Avalos-Navarro, Alma D. Paz-González, Adriana Moreno-Rodríguez, Benjamín Nogueda-Torres, Gildardo Rivera

**Affiliations:** 1 Laboratorio de Biotecnología Farmacéutica, Centro de Biotecnología Genómica, Instituto Politécnico Nacional, Reynosa 88710, Mexico; doomadv@hotmail.com (D.M.-Á.); al.gonzalez.gonzalez88@gmail.com (A.G.-G.); apazg@ipn.mx (A.D.P.-G.); 2 Departamento de Ciencias Médicas y de la Vida, Centro Universitario de la Ciénega (CUCIÉNEGA), Universidad de Guadalajara, Av. Universidad 1115, Lindavista, Ocotlán 47820, Mexico; guadalupe.avalos5337@academicos.udg.mx; 3 Laboratorio de Estudios Epidemiológicos, Clínicos, Diseños Experimentales e Investigación, Facultad de Ciencias Químicas, Universidad Autónoma “Benito Juárez” de Oaxaca, Avenida Universidad S/N, Ex Hacienda Cinco Señores, Oaxaca 68120, Mexico; arimor@hotmail.com; 4 Departamento de Parasitología, Escuela Nacional de Ciencias Biológicas, Instituto Politécnico Nacional, Mexico City 11340, Mexico; bnogueda@yahoo.com

**Keywords:** *Trypanosoma cruzi*, drug repositioning, FDA, TBP, in silico, in vitro

## Abstract

**Background:** Parasitic diseases, particularly Chagas disease caused by *Trypanosoma cruzi*, primarily affect developing countries but are now spreading to wealthier nations due to changing migration patterns. With approximately 8 to 9 million cases annually and a rise in drug resistance and side effects, there is an urgent need for new therapeutic approaches. **Objectives:** This study aimed to identify potential pharmacological compounds to target the TATA Binding Protein (TBP) of *T. cruzi*. **Methods:** Over eleven thousand FDA-approved pharmacological compounds were analyzed using in silico methods, including homology modeling, molecular docking, and molecular dynamics simulations. In addition, in vitro assays were conducted to assess the trypanocidal activity of promising candidates against *T. cruzi* epimastigotes and their selectivity toward macrophage J774.2. **Results:** Two compounds, DB00890 and DB07635, emerged as promising candidates, demonstrating significant potential against *T. cruzi* TBP. Compound DB00890 had trypanocidal activity against *T. cruzi* epimastigotes, with IC_50_ values of 70.4 µM (SI 2.84) and 37.3 µM (SI 5.36) for the NINOA and A1 strains, respectively. **Conclusions:** Our findings suggest DB00890 is a promising candidate for the development of new agents against Chagas disease, with the potential for targeted therapies that minimize side effects. These results provide a strong foundation for further research into novel treatments for parasitic diseases caused by *T. cruzi*.

## 1. Introduction

Parasitic diseases caused by trypanosomatids such as *Trypanosoma cruzi* put significant pressure on the healthcare systems of the countries where this disease is endemic [1,2,3]. Moreover, current migration patterns, driven by various factors, have spread this parasitosis to areas where it is not commonly found [4]. Parasitic infections caused by members of the Trypanosomatidae family, such as *T. cruzi*, impose a substantial economic burden, particularly on Ibero-American countries [5]. The infection caused by this parasite can be fatal or lead to significant physical impairment. While several treatment options are available, it is important to note that some of these medications may cause side effects that vary in intensity from minor to serious [6,7]. Furthermore, several studies have documented a rising incidence of resistance to the drugs commonly used to treat this disease [7,8]. Untimely intervention in these parasitic infections, combined with compromised immune systems, makes these disease factors contribute to mortality and morbidity, particularly in the most impoverished areas [5,6,7].

American trypanosomiasis or Chagas disease is transmitted by the *T. cruzi* parasite through the bite of a triatomine insect. Estimates indicate that there are 8–9 million cases reported worldwide. Most of these cases are reported in Latin America, with Brazil, Colombia, and Peru being highly endemic areas [5]. This parasitosis primarily affects vulnerable groups living in areas with precarious health systems. Children and the immunocompromised population are particularly susceptible to developing severe forms of this disease. American trypanosomiasis and twenty other diseases have been categorized as Neglected Tropical Diseases (NTD) by the World Health Organization (WHO) since the beginning of this century [9,10,11].

This protist presents a complex life cycle, which includes a vector, a triatomine insect, and a vertebrate host, generally a mammal; the life cycle can be divided into three stages: epimastigote, amastigote, and trypomastigote [12,13]. The main drugs to combat this disease are directed against the amastigote stage, which is the intracellular and reproductive form of the parasite within the host, and the frontline medications for treating this infection are nitroimidazole and nitrofuran derivatives, both of which may have mild to severe adverse effects [14,15]. These two drugs can generate reactive oxygen species that, in turn, cause damage to the parasite’s DNA and chemically modify lipids and proteins; it is worth remembering that *T. cruzi* does not possess catalase enzymes and has peroxidases that are not very effective [16,17].

The drugs currently used to treat *T. cruzi* infections were developed over 50 years ago, leading to a rise in drug resistance. Additionally, the reported side effects highlight the urgent need to develop new medications and explore alternative therapeutic approaches to more effectively combat this disease, which affects countless individuals across the globe.

Transcription factors have recently emerged as a promising target for treating parasitic diseases, primarily due to the divergence in amino acid composition compared to complex eukaryotic organisms and the relatively limited genetic variability observed in this type of protein (e.g., transcription factors). These factors play a critical role in regulating gene expression, making them an attractive target for novel therapeutic approaches to disrupt essential biological processes in the parasite without affecting the host [18,19,20,21,22,23,24,25].

The TATA-box recognizing factor (TBP) is a widely conserved gene expression regulator crucial for initiating transcription in eukaryotes. It binds to the TATA box in gene promoters. It is essential for the activation of all three eukaryotic RNA polymerases: RNA polymerase I (for rRNA), RNA polymerase II (for mRNA and snRNAs), and RNA polymerase III (for tRNA and other small RNAs) [26,27]. TBP regulates gene expression across various biological processes [26,27]. Given the marked divergence in the amino acid composition of the TATA-box recognizing proteins found in basal protists [18,21,23,24,25], along with the low mutation rates observed in these proteins and other transcription factors [22,23], TBP is proposed as a promising target for specific therapies against disease transmitted by the *T. cruzi* parasite. This is supported by numerous crystallographic studies that reveal the extensive interaction of TBP’s surface with additional components of the transcriptional machinery [20]. Therefore, interfering with such molecular associations by targeting the interface regions among proteins may hinder the assembly of the transcriptional pre-initiation machinery. This would interfere with the essential assembly required for transcription, suppressing gene expression by all three RNA polymerases. Such an approach could effectively target the transcriptional machinery of parasitic organisms, potentially offering a new strategy for therapeutic intervention. CX-5461, an investigational cancer therapeutic, serves as an example, as it targets ribosome biogenesis by inhibiting ribosomal RNA synthesis [28,29]. Unlike other inhibitors, CX-5461 does not affect the formation of the pre-initiation complex or the recruitment of transcription factors but irreversibly blocks RNA polymerase I from releasing the promoter [29]. This results in a persistent and unproductive transcription initiation complex, leading to nucleolar stress, DNA damage, and cellular senescence [29]. The drug shows promise in early trials for treating various cancers and highlights a new approach to developing chemotherapeutic strategies [28,29]. Furthermore, using small molecules to inhibit interactions with other transcription factors could be highly detrimental to parasites. For instance, Negative Cofactor 2 (NC2) acts as a negative regulator of TBP by blocking the binding of TFIIA and TFIIB, which are essential for initiating transcription [30]. A small molecule capable of tightly engaging the NC2 motif may obstruct the formation of the pre-initiation complex by preventing the association of TFIIA and TFIIB. This interference could impair the transcription process crucial for the parasite’s survival and proliferation, potentially leading to its death. This approach underscores the potential of targeting specific protein–protein interactions in the transcription machinery as a strategic method to develop effective treatments against parasitic diseases. Strategies for inhibiting protein interactions are detailed in the works of Lambert et al. (2018) and Walters et al. (2021), which explore their potential applications in treating both cancer and parasitic diseases caused by protists [24,31]. These studies provide insights into how targeting specific protein–protein interactions can be leveraged to disrupt critical biological processes in this disease. By focusing on inhibiting key transcription factors and other essential proteins, these strategies offer new avenues for developing therapies that could effectively combat cancer and parasitic infections [24,31]. To address this issue, we sought to evaluate more than 11,000 drug-like molecules curated from the FDA-approved chemical library to determine their efficacy and investigate their use in treating Chagas disease.

This study used molecular modeling techniques, including homology modeling, molecular docking, and molecular dynamics, to assess the inhibitory effects of 7969 FDA-sourced compounds on the *T. cruzi* TBP. We focused on analyzing the interactions of these compounds, which passed the Lipinski Rule of Five filter, with the NC2 motif of TBP from *T. cruzi* (*Tc*TBP) and evaluated their effects on human TBP (*Hs*TBP). This analysis identified compounds with the highest selectivity for *Tc*TBP compared to *Hs*TBP. To validate the in silico prediction, the compounds selected were evaluated using in vitro assays.

## 2. Results

### 2.1. Multiple Sequence Alignments

Given the lack of experimentally resolved tertiary structures for the TBPs of *T. cruzi*, we adopted an alternative approach by analyzing crystallographic structures from a broad range of eukaryotic species. These species belong to various taxonomic groups—including animals, plants, and fungi—which together provide a diverse structural data set. This method enabled us to carry out a detailed structural alignment that served as a reference framework for the sequences of *Tc*TBP, whose structure remains unresolved. By utilizing the resolved crystal structures (1JFI, 1QNA, 1NH2, and 3OCI), we could accurately delineate the DNA-interacting region within the *Tc*TBP.

**Figure 1:**
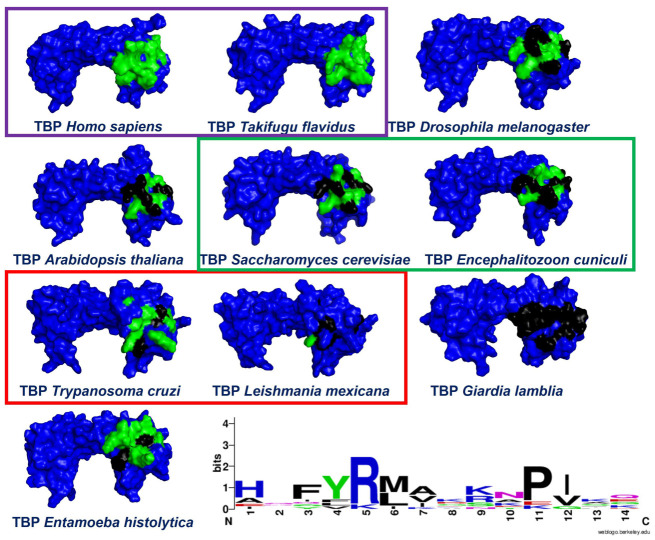
Illustrates eukaryotic TBPs, highlighting the 14 amino acid residues forming the NC2 motif. Residues conserved for *Hs*TBP are marked in green, while non-conserved residues are depicted in black. In the lower-right corner, the WebLogo diagram shows the degree of residue preservation across the 14 amino acids forming the NC2 region. The purple box shows the TBPs from animals; note that the NC2 motif is entirely conserved. The green box shows the TBPs from organisms in the Fungi kingdom; note the presence of amino acid residues that differ from those in human TBP. Finally, the red box displays the TBPs from *T. cruzi* and the related protist, *Leishmania mexicana*; note the significant number of changes in the residues that make up the NC2 motif and that several residues are buried within the protein structure.

Additionally, we included TBP protein sequences from various eukaryotic taxonomic groups in our multiple alignment of sequences (see Supplementary Materials Figure S1). This allowed us to evaluate the preservation of residues comprising the NC2-associated region. Interestingly, a reduced level of residue conservation at the interaction interface indicates a greater likelihood of discovering pharmacologically active compounds capable of interfering with the accurate assembly of the transcriptional machinery. As previously mentioned, the three-dimensional alignment provided a framework for aligning the rest of the sequences for which 3D conformations have not been resolved. This approach is grounded in the idea that the tertiary structure of a protein tends to be more preserved over evolutionary time than the linear sequence of its amino acids. This holds particularly true in the case of evolutionarily conserved protein families, where the polypeptides exhibit analogous functions and structural features due to their shared ancestral origins. Moreover, recent studies have demonstrated that even minor structural variations within these conserved domains can result in significant functional diversity, underscoring the importance of structure-based approaches in comparative protein analysis [32,33,34]. In addition, the amino acid residues known to interact with NC2 were marked in the different three-dimensional models shown in Figure 1.

Notably, the residues that mediate the interaction with NC2 remain strongly conserved across vertebrate species, consistent with earlier findings on the conservation of TBPs in this animal group [23]. This high level of conservation reflects the essential role of these residues in maintaining proper transcriptional function [23]. As evolutionary distance grows, the number of altered residues increases as well. For example, in *A. thaliana*, *E. cuniculi*, and *S. cerevisiae*, 7 of the 14 residues involved in the interaction with NC2 remain conserved. Interestingly, in *E. histolytica*, *L. mexicana*, and *T. cruzi*, only 3 of these 14 residues are preserved. This reduction in conservation highlights the evolutionary divergence of these species and suggests that changes in these residues may impact the functionality of TBPs in these more distantly related organisms. Finally, a noteworthy case is that of *Giardia lamblia*, where all 14 residues forming the NC2 motif are altered. It was recently reported that *G. lamblia* TBP is susceptible to inhibition by small molecules without affecting human TBP (*Hs*TBP) [25]. Lastly, it is important to highlight that in *T. cruzi,* specific residues that make up the NC2 motif seem buried within the protein structure, as shown in Figure 1.

### 2.2. In Silico Analysis of TcTBP and HsTBP

**Figure 2:**
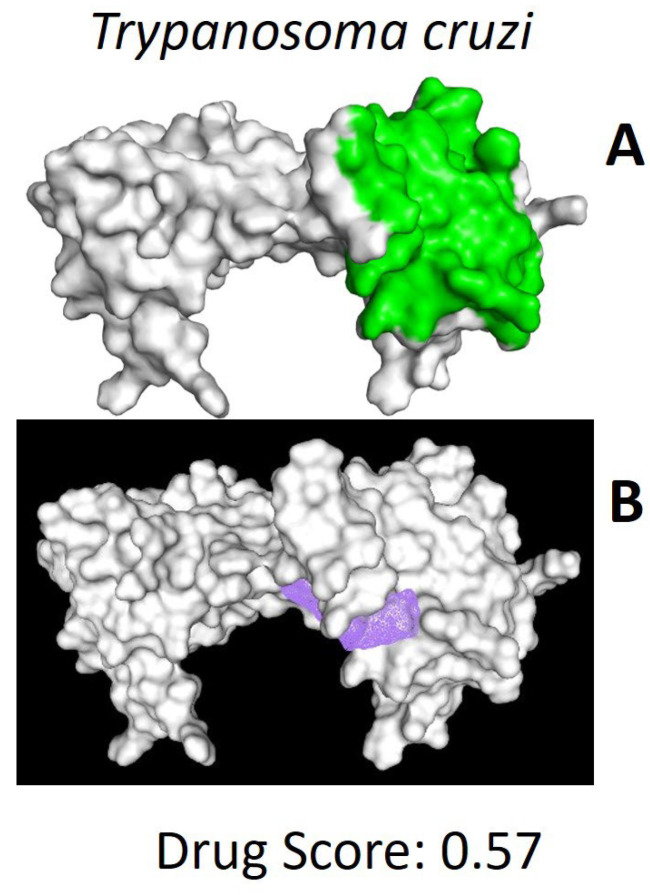
Possible ligand-binding sites. (**A**) Results of blind docking experiments. (**B**) Potentially druggable sites identified by the DoGSiteScorer Binding Site server. For *T. cruzi*, the second-most promising druggable site is presented, indicated by its drug score. The NC2 site is also mentioned in the literature as a potential therapeutic target for parasites, including protozoa such as *Plasmodium falciparum* and *Entamoeba histolytica*.

After locating the putative ligand-binding region (see Figure 2), computational modeling approaches were employed to estimate the interactions of compounds previously identified through screening of the DrugBank database. This approach enabled the identification of compounds with the highest affinity and selectivity for the TBP binding site in *T. cruzi*.

#### 2.2.1. TcTBP Molecular Docking

**Figure 3:**
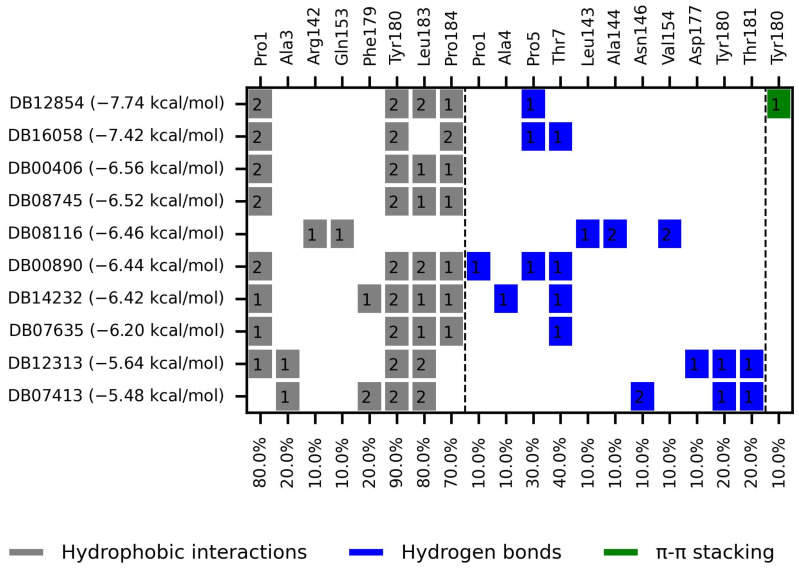
The binding profile of the ten leading compounds shows higher selectivity for *Tc*TBP than for *Hs*TBP. On the *x*-axis, the percentage of interactions of each amino acid residue with the ligand is shown. At the same time, on the *y*-axis, the interacting compound is displayed along with its binding free energy.

**Figure 4:**
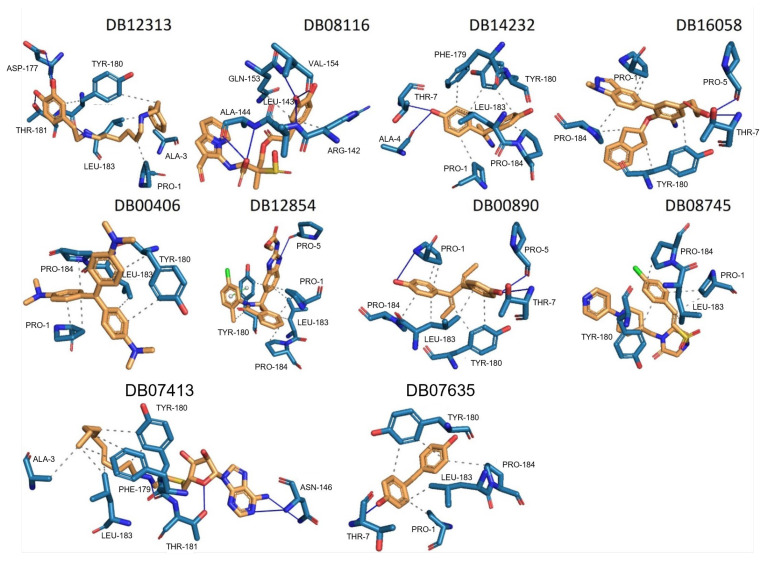
Illustrates the interactions between amino acid residues of the NC2 motif and various ligands. The ten compounds with the highest selectivity index for *Tc*TBP in comparison to *Hs*TBP are displayed.

We selected the top ten compounds based on binding free energies below −5.4 kcal/mol and negative selectivity indices, indicating stronger predicted binding to *Tc*TBP over *Hs*TBP. The ten compounds with the highest selectivity for *Tc*TBP over *Hs*TBP were DB00406, DB12854, DB08116, DB16058, DB07413, DB12313, DB08745, DB00890, DB14232, and DB07635 (Table 1 and Figure 3 interaction chart and Figure 4 interactions between the NC2 motif and ligands).

DB12313 (dopexamine) exhibited a binding free energy (BFE) of −5.645 kcal/mol and a selectivity value of −1.367 relative to *Hs*TBP. It interacted hydrophobically with Pro1, Ala3, and Leu183 and formed hydrogen bonds with Asp177 and Thr181. Tyr180 was involved in both hydrophobic interactions and hydrogen bonding.

DB08116 exhibited a binding free energy (BFE) of −6.465 kcal/mol and a selectivity value of −1.242 relative to *Hs*TBP. The compound formed two hydrophobic interactions with Arg142 and Gln153 and established three hydrogen bonds with Leu143, Ala144, and Val154.

DB14232 (deacetylbisacodyl) exhibited a binding free energy (BFE) of −6.422 kcal/mol and a selectivity value of −1.086 relative to *Hs*TBP. The compound formed five hydrophobic interactions with Pro1, Phe179, Tyr180, Leu183, and Pro184 and established two hydrogen bonds with Ala4 and Thr7.

DB16058 (AK106-001616) exhibited a binding free energy (BFE) of −7.416 kcal/mol and a selectivity value of −1.083 relative to *Hs*TBP. The compound formed three hydrophobic interactions with Pro1, Tyr180, and Pro184 and established two hydrogen bonds with Pro5 and Thr7.

DB00406 (gentian violet) exhibited a binding free energy (BFE) of −6.561 kcal/mol and a selectivity value of −1.046 relative to *Hs*TBP. The compound formed four hydrophobic interactions with Pro1, Tyr180, Leu183, and Pro184 and did not form any hydrogen bonds with surrounding residues. It is worth mentioning that previous studies have shown the effect of gentian violet at a concentration of 250 µg/mL together with ascorbic acid at 2 mg/mL and photo radiation (75 W) for six hours on the sterilization of blood samples [37]. Furthermore, recent research suggests that gentian violet could be repurposed as a potential therapeutic approach in the treatment of ovarian cancer [43]. Moreover, its ability to inhibit the growth of cancer cells has been demonstrated in experiments with mice carrying xenotransplants of human hepatocellular carcinoma [44]. Finally, structural derivatives of gentian violet have demonstrated anti-trypanosomal properties by blocking the proline transporter enzyme, which is vital for this parasite [45].

DB12854 (BMS-908662) exhibited a binding free energy (BFE) of −7.741 kcal/mol and a selectivity value of −1.035 relative to *Hs*TBP. It formed hydrophobic interactions with Pro1, Leu183, and Pro184 and established a hydrogen bond with Pro5. Tyr180 was involved in both the hydrophobic interaction and π–cation-like stacking.

DB00890 (dienestrol) exhibited a binding free energy (BFE) of −6.376 ± 0.067 kcal/mol and a selectivity value of −0.967 ± 0.067 relative to *Hs*TBP. It formed hydrophobic interactions with Tyr180, Leu183, and Pro184 and established hydrogen bonds with Pro5 and Thr7. Pro1 was involved in both hydrophobic contact and hydrogen bonding. It is worth mentioning that a decrease in the severity of the infection caused by protist parasites, including trypanosomes, in response to certain estrogens (dienestrol is a synthetic, nonsteroidal estrogen) has been reported. Furthermore, a decrease in the load of trypomastigotes in the bloodstream has been reported in response to the administration of dehydroepiandrosterone (DHEA) [39,40,46].

DB08745 had a binding free energy (BFE) of −6.521 kcal/mol and a selectivity value of −0.985 for *Hs*TBP. This compound showed four hydrophobic-type interactions with the amino acid residues Pro1, Tyr180, Leu183, and Pro184. Additionally, the compound does not form any hydrogen bonds with residues.

DB07413 exhibited a binding free energy (BFE) of −5.476 kcal/mol and a selectivity value of −1.035 relative to *Hs*TBP. It formed hydrophobic interactions with Ala3, Phe179, and Leu183 and established hydrogen bonds with Asn146 and Thr181. Tyr180 was involved in both hydrophobic interactions and hydrogen bonding.

DB07635 (4,4′-Dihydroxybenzophenone) had a binding free energy (BFE) of −6.180 ± 0.018 kcal/mol and a selectivity value of –0.956 ± 0.018 for *Hs*TBP. This compound showed four hydrophobic-type interactions with the amino acid residues Pro1, Tyr180, Leu183, and Pro184. Additionally, the compound forms one hydrogen bond with residue Thr7. It is worth mentioning that this compound (4,4′-Dihydroxybenzophenone) inhibits sterol biosynthesis, which is detrimental to Trypanosoma and various species of Leishmania [42].

#### 2.2.2. TcTBP Molecular Dynamics

For the molecular dynamics simulations, we selected economically viable compounds and those that are competitive with existing drugs, as these diseases predominantly affect low-income regions. The compounds analyzed included DB00890 and DB07635 for *T. cruzi*.

**Figure 5:**
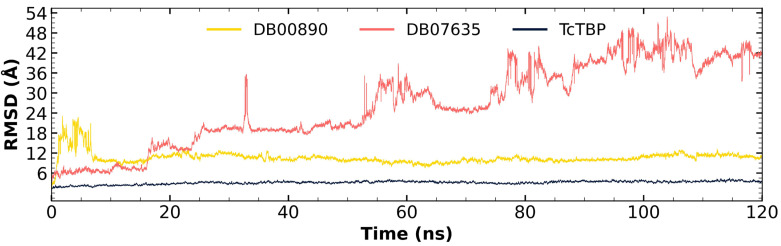
RMSD plot for *Tc*TBP apo-protein and in complex with FDA-approved compounds DB00890 and DB07635.

In the case of *Tc*TBP, it is worth noting that the compound DB00890 successfully stabilized within the first 8 nanoseconds of the simulation and reached equilibrium and remained stable at around 10–12 Å, which is considered acceptable for systems of this size, maintaining an average fluctuation of 10.59 Å throughout the trajectory (refer to Figure 5). This behavior suggests the potential of this compound as a *Tc*TBP inhibitor. Although it exhibited fluctuations at the beginning of the dynamics simulation, it ultimately attained a stable pose, which it maintained until the end of the simulation. Secondly, although DB07635 showed high RMSD values throughout the simulation, which may suggest reduced structural stability, its trajectory did not indicate complete dissociation or irreversible unfolding. Instead, the RMSD profile suggests that the ligand remains associated with *Tc*TBP despite its conformational flexibility. Considering its favorable binding free energy and sustained interaction over the simulation time, DB07635 remains a viable candidate for experimental validation, as molecular flexibility does not necessarily preclude biological activity and could reflect an induced-fit mechanism.

Through an analysis of the RMSF graph, we evaluated the fluctuations in the different TBPs, both in their isolated form and when complexed with various compounds.

**Figure 6:**
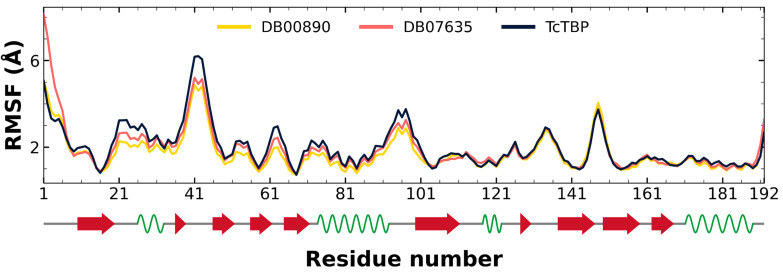
RMSF graphs for *Tc*TBP apo-protein and FDA-approved DB00890 and DB07635. Secondary structure elements of *Hs*TBP are annotated with red arrows (β-sheets), green sinusoidal lines (α-helices), and gray lines (unstructured regions).

In the case of *Tc*TBP, it can be observed that the two compounds in complex with the protein exhibit RMSF profiles like that of the protein alone, with higher fluctuations at the terminal ends, which are associated with unstructured elements (refer to Figure 6).

**Figure 7:**
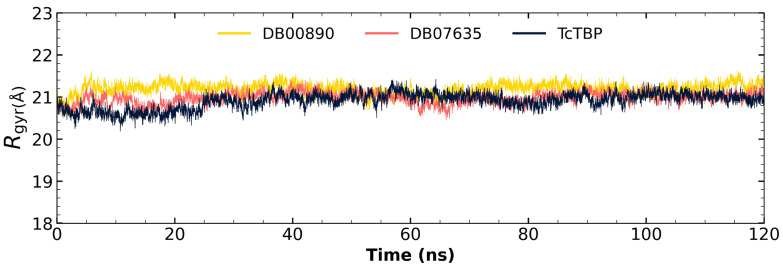
Gyration radius graphs for *Tc*TBP apo-protein and in complex with potential inhibitors DB00890 and DB07635.

Differences in fluctuations between the maximum and minimum values for the radius of gyration are observed, with values of 1.8396 Å. These findings suggest that the diverse TBP models maintain a compact structure throughout the simulation, both in their isolated form and in complex with ligands (see Figure 7). As a reference, the radius of gyration for *Hs*TBP (PDB ID 1JFI) in its isolated form shows a fluctuation difference of 1.7507 Å.

**Figure 8:**
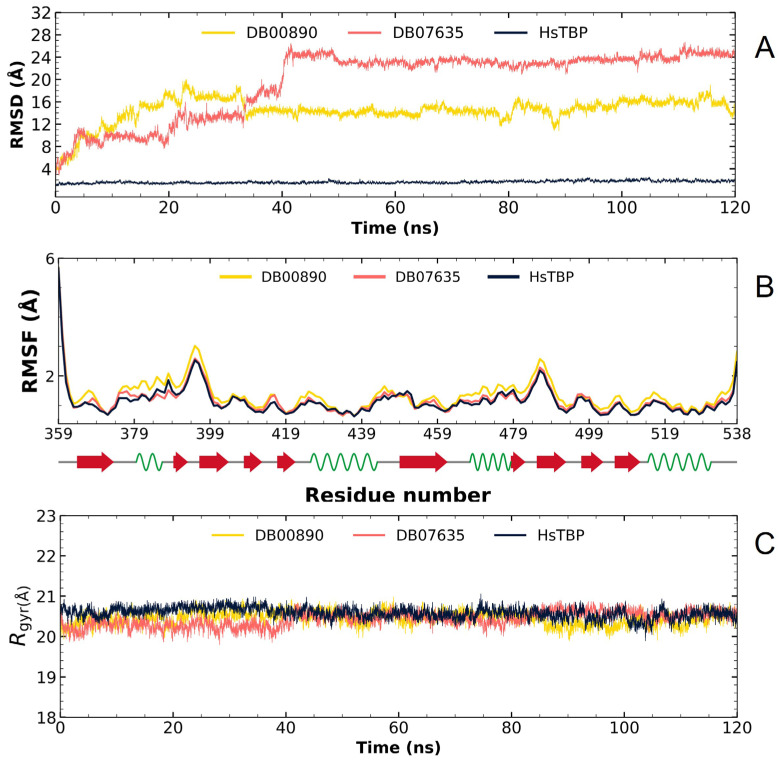
Graphs derived from the molecular dynamics analysis of *Hs*TBP: (**A**) RMSD profiles for the apo form and complexes with FDA-approved compounds DB00890 and DB07635. (**B**) RMSF profiles for the apo form and ligand-bound states; secondary structure elements of *Hs*TBP are annotated with red arrows (β-sheets), green sinusoidal lines (α-helices), and gray lines (unstructured regions). (**C**) Radius of gyration plots for the apo-protein and its complexes with DB00890 and DB07635.

Based on the RMSD analysis, it is evident that DB00890 and DB07635 display fluctuations initially when bound to *Hs*TBP, which are then stabilized after about 40 ns, as illustrated in Figure 8A, suggesting that they may act potentially as *Tc*TBP and *Hs*TBP inhibitors, yet the in vitro results seem to favor trypanocidal activity over cytotoxic activity. The RMSF graph analysis shows minimal fluctuation across the protein structure, as depicted in Figure 8B. The gyration radius graph in Figure 8C also indicates that the protein remains stable throughout the simulation, both alone and in complex. Consequently, the fluctuations observed in the RMSD graph are attributed to the reduced stability of the two compounds within *Hs*TBP, leading to greater inhibitory selectivity for *Tc*TBP.

In summary, these findings support the hypothesis that the evaluated compounds DB07635 and DB00890 could have effective potential for repositioning against *Tc*TBP, with promising implications for developing selective inhibitors. In Supplementary Figure S2, key interacting residues with DB00890 are highlighted alongside the secondary structure of *Tc*TBP, contextualizing flexibility and the binding sites.

### 2.3. In Vitro Assays

Compounds DB07635 and DB00890 were acquired from a chemical supplier (Molport) and assessed for their efficacy against epimastigotes of the Mexican NINOA and A1 strains of *T. cruzi* and cytotoxicity against macrophage J774.2. Only derivative DB00890 had trypanocidal activity under 200 µM, with IC_50_ values of 70.40 and 37.25 μM against the NINOA and A1 strains, respectively (Table 2).

## 3. Discussion

### 3.1. In Silico Evaluation

While transcription factors were once thought to be impossible to target with drugs, recent findings suggest they could be promising targets for treating various conditions, including cancer [31]. There has even been discussion about their potential use in treating parasitic diseases [24]. Even though TBP is crucial for transcription and nearly all its surface engages with other transcription factors or DNA, it has not been thoroughly explored as a potential therapeutic target [20]. There are three instances where TBP has been recognized as a target. The first involves an indirect approach, where the inhibition of kinases that phosphorylate TBP alters its binding affinity to the gene promoter region [47,48]. The second case includes various examples, particularly in cancer research, in which DNA-reactive alkylating compounds interfere with the accurate recruitment of TBP at the promoter site of target genes [31]. These agents interact with the concave area of TBP’s distinctive saddle-shaped structure [31]. Finally, in a study we recently published, we proposed TBP from *G. lamblia* (*Gl*TBP) as a potential therapeutic target. In this study, the NC2 motif was identified as a potential drug-binding site that could interfere with the transcription process [25]. Additionally, it is important to note that recent research by Santiago et al. [20] has identified distinct areas on the outward-curved surface of the TBP protein in protozoans like *E. histolytica* and *P. falciparum* as promising sites of therapeutic action. This is due to their unique structural traits and differences in protein sequence composition. This finding aligns with our recent study that suggests that the TBP from *G. lamblia* (*Gl*TBP) could also be a viable target, mainly focusing on the NC2 motif as a potential drug-binding site that might impair the transcription process. It has been proposed that using small molecules to inhibit protein–protein or protein–DNA interactions could impair transcription, potentially harming the parasite [24,31]. Consequently, TBPs with significant sequence variation are viable candidates for therapeutic targeting. For example, the TBP from *Trichomonas vaginalis* shows such considerable divergence that it fails to function in other organisms, such as *Saccharomyces cerevisiae*, as demonstrated by complementation assays [49]. This highlights the potential of leveraging these differences to develop specific treatments without affecting other species. Our analysis shows that the NC2 motif in the TBP of *T. cruzi* exhibits significant sequence divergence. Only three of the fourteen amino acid residues interacting with NC2 are conserved in *Tc*TBP (Figure 1). Furthermore, there is a noticeable morphological alteration in the cavity, which might allow small molecules to disrupt the proper assembly of the pre-initiation complex without impacting *Hs*TBP (see Figure S3 Supplementary Data). This approach is similar to what Santiago et al. suggest for the TBP of *E. histolytica* and *P. falciparum* [20]. Such modifications could offer a strategic advantage in developing targeted therapies.

In this study, we evaluated more than 11,000 compounds from DrugBank to find potential inhibitors of the TBP protein in the medically important *T. cruzi* protist. For the *Tc*TBP protein, we found that the compound DB00890 (dienestrol) shows potential as an inhibitor, with a binding energy of −6.376 ± 0.067 kcal/mol and a selectivity of −0.967 ± 0.067 relative to *Hs*TBP. This compound also forms hydrophobic interactions with residues P1, Y180, L183, and P184 and hydrogen bonds with P1, P5, and T7. Supplementary Figure S4 shows that some of these hydrogen bonds are not sustained over time, highlighting differences between docking predictions and dynamic behavior. This suggests that the initial interactions may reflect a transient binding mode, potentially compatible with an induced-fit mechanism. Additionally, it has been reported that certain estrogens, such as dienestrol, and dehydroepiandrosterone (DHEA) can reduce the severity of infections caused by protist parasites, including trypanosomes, and lower the number of trypomastigotes in the bloodstream [39,40]. Molecular dynamics simulations suggest a relatively stable binding of the formed complex, raising the possibility that this compound could be subjected to in vitro assays.

While DB00890 showed promising selectivity toward *Tc*TBP and moderate trypanocidal activity, it remains less potent than current standard treatments such as benznidazole and nifurtimox. Therefore, further optimization and potency improvement are required before considering clinical translation, although its distinct binding profile makes it a valuable starting point for future drug development.

In summary, the primary interactions observed in this complex and among the top ten with the highest selectivity index are hydrophobic interactions, followed by hydrogen bond interactions. Specifically, Y180 is found in 90% of the complexes analyzed, while L183 and P1 appear in 80% of the protein–ligand complexes and P184 in 70% (see Figure 3). Both hydrophobic interactions and hydrogen bonds are essential for the structure and stability of these complexes.

The set of ten leading compounds exhibiting the highest selectivity index for *Tc*TBP shows that only a few residues identified in our structural alignment are reported to interact with the top ten compounds with the best selectivity for *Tc*TBPs. This is possibly because these sequences have more extended NC2 binding domains, and some of the residues identified in our structural alignment appear buried within the protein structure, as illustrated in Figure 1. The residues identified in our structural alignment that exhibit interactions with *Tc*TBP include P184, forming six hydrophobic interactions and one hydrogen bond, T181, forming two hydrogen bonds, and L143, A144, and R142, with the first two each forming a hydrophobic interaction and the latter a hydrogen bond. Regarding the residues that show the highest number of interactions in *Tc*TBP, we have Y180 with twelve interactions, predominantly of the hydrophobic type, followed by P1 with nine interactions, primarily of a hydrophobic nature, and L183 with eight interactions of a hydrophobic type. It is worth mentioning that amino-terminal residues are involved in the reported interactions in *Tc*TBP. This is evident in our RMSF analysis, as shown in Figure 6, where the first ten residues in this protein exhibit high fluctuations primarily due to their disordered nature and proximity to the NC2 motif.

It is worth noting that the selected compounds were prioritized based on their dual mode of action—binding to *Tc*TBP and targeting parasite-specific pathways, such as the infection-reducing effects reported for DB00890 against trypanosomatids and the inhibition of sterol biosynthesis by DB07635, a metabolic pathway essential for parasite viability but absent in humans. This prioritization also considered the urgent public health need for new treatments for Chagas disease, a WHO-recognized Neglected Tropical Disease (NTD) that disproportionately affects vulnerable populations with limited access to healthcare. In this context, repurposing affordable and already approved drugs represents a practical strategy to accelerate therapeutic development. In addition to availability and cost, the selection of DB00890 (dienestrol) and DB07635 (4,4′-Dihydroxybenzophenone) was supported by their favorable safety and pharmacological profiles. DB00890 is a synthetic nonsteroidal estrogen with a well-established history of clinical use. DB07635 has low acute toxicity and has been evaluated for use in cosmetic and industrial applications. Both compounds also meet general drug-likeness criteria, supporting their suitability for in vitro testing. Finally, our structural alignment and comparative analysis revealed that the NC2 motif in *T. cruzi* TBP exhibits substantial sequence divergence compared to human and other eukaryotic TBPs, as illustrated in Figure 1. Notably, only three of the fourteen NC2 residues are conserved in *Tc*TBP, and several non-conserved positions are structurally buried or exhibit distinct physicochemical properties. These structural differences likely reshape the binding cavity, providing a molecular basis for the selective binding of small molecules to *Tc*TBP without affecting human TBP. This observation supports the selectivity indices obtained in our docking analyses and aligns with previous reports proposing TBP divergence in protists as a therapeutic opportunity. Such divergence enhances the potential for parasite-specific targeting and reduces the likelihood of off-target effects in the host.

### 3.2. In Vitro Evaluation

The trypanocidal activity observed for DB07635 and DB00890 shows that on the one hand, DB07635 can be considered as ineffective as a trypanocidal agent, as it shows IC_50_ values over 200 µM against both tested *T. cruzi* strains. On the other hand, DB00890 is a moderately active compound as a trypanocidal agent, with IC_50_ values of 70.4 and 37.3 µM for the NINOA and A1 strains, respectively. Compound DB00890 is 2.3-fold and 10-fold less active than benznidazole and nifurtimox, respectively, against the NINOA strain and about 2-fold less active than nifurtimox against the A1 strain; however, it is slightly better than benznidazole against the A1 stain. Both DB07635 and DB00890 were found to be less toxic toward macrophages than the reference drugs. The selectivity index of DB00890 toward *T. cruzi* over macrophages is 2.86-fold and 5.36-fold against NINOA and A1, respectively, suggesting that this compound may be further explored as a candidate for the development of a new trypanocidal agent.

## 4. Materials and Methods

### 4.1. Download of Protein Sequences

The amino acid sequence for *T. cruzi* (*Tc*TBP) was obtained from TriTrypDB (https://tritrypdb.org, accessed on 15 December 2024)). To perform structural alignment, resolved structures of eukaryotic TBPs were sourced from the RCSB PDB database. This enabled us to define the carboxyl-terminal domain of the sequences for evaluation and retrieve TBP 3D conformations from *Homo sapiens* (*Hs*TBP; PDB ID: 1JFI), *Arabidopsis thaliana* (PDB ID: 1QNA), *Saccharomyces cerevisiae* (PDB ID: 1NH2), and *Encephalitozoon cuniculi* (PDB ID: 3OCI). Moreover, other eukaryotic amino acid sequences were fetched from NCBI using the BlastP tool with the 1JFI sequence as the query (https://blast.ncbi.nlm.nih.gov/Blast.cgi, accessed on 18 January 2025). These additional sequences include those from *Entamoeba histolytica* (XP_654935), *Drosophila melanogaster* (NP_523805), and *Takifugu flavidus* (XP_056871520). These sequences contributed to a more robust alignment and facilitated examination of conserved residues within the NC2 region.

### 4.2. Multiple Sequence Alignments

The three-dimensional conformation comparison was conducted using the PDBeFold server (https://www.ebi.ac.uk/msd-srv/ssm/, retrieved on 20 January 2025) from the Protein Data Bank Europe (PDBe) for the four structures obtained from the RCSB PDB (1JFI, 1QNA, 1NH2, and 3OCI). This multiple sequence alignment (MSA) served as a basis for aligning the remaining amino acid chains, including those from TriTrypDB and NCBI. The second alignment was performed using the Clustal omega algorithm, informed by the initial structural alignment. After completing this alignment, we defined the protein sequences’ DNA-binding domain (C-terminal) from organisms whose TBP structures had not been experimentally resolved. Then, we predicted their 3D conformation with the AlphaFold2 server [50,51]. After all the 3D conformations were generated, we identified and highlighted the residues implicated in recognizing Negative Cofactor 2 (NC2) across both crystallographically resolved and computationally modeled structures, using the 1JFI entry as a reference—the sole TBP–NC2 complex currently available in the RCSB PDB.

### 4.3. Identification of the Ligand Interaction Region

Various approaches were employed to estimate potential binding sites for *Tc*TBP. The DoGSiteScorer platform for ligand interaction prediction was utilized for this purpose [52]. Additionally, blind docking experiments were conducted between the aforementioned proteins and the 7969 compounds under evaluation [53,54]. A literature review also identified the NC2 interaction region as a promising candidate for therapeutic blockade [20,21].

### 4.4. Docking Simulation

#### 4.4.1. Receptor Preparation

The *Hs*TBP structure (PDB ID: 1JFI) was processed for docking simulations using the Chimera molecular modeling system [55]. Initially, ligands and solvent molecules were excluded from the PDB entry 1JFI corresponding to the human TBP available in the RCSB PDB. Subsequently, the tridimensional structure of *T. cruzi* (retrieved from TriTrypDB) and the processed crystallographic model 1JFI underwent protonation, and their partial charges were calculated using the Gasteiger method implemented in AutoDock Tools v.4.2 [56]. Both structures were ultimately exported in PDBQT format.

#### 4.4.2. Ligand Configuration for Docking Simulations

We collected 11,584 compounds from DrugBank (https://go.drugbank.com/, accessed on 23 January 2025), which included FDA-authorized compounds initially in SDF format. Each was transformed into MOL format with OpenBabel v2.4.1 and subjected to filtering according to the Lipinski criteria [57]. After this filtration, 7969 compounds were selected. These molecules were further processed using MGLTools (version 1.5.6), where their structures were optimized through energy minimization, the addition of polar hydrogen atoms, and conversion into PDBQT format.

#### 4.4.3. Grid Parameter Configuration

A 3D docking grid was established based on the fourteen residues that engage with NC2, identified based on the X-ray crystallographic model of TBP bound to NC2 (PDB ID: 1JFI). The locations of these amino acids in the *Trypanosoma cruzi* sequence were deduced via tertiary structure comparison. The docking region was configured with specific coordinates and dimensions for *Tc*TBP. The coordinates along the *X*, *Y*, and *Z* axes were set to −14.952, −13.115, and 6.944, in that order, with each dimension spanning 32 Å.

Next, molecular docking analysis was performed in triplicate using AutoDock Vina (version 1.2.3) with an exhaustiveness value of 64, generating nine binding modes (default setting) and obtaining pose 1 as the best-ranked result [58,59], with each *Tc*TBP and *Hs*TBP as the receptor for the 7969 previously prepared compounds. We then compared the binding free energy (BFE) of each compound with *Hs*TBP against their values with *Tc*TBP to identify the 10 compounds with the highest selectivity for the *Trypanosoma cruzi* TBP, as reflected by their most favorable BFE values. PLIP (Protein–Ligand Interaction Profiler) (v. 2.2.2) was used to characterize the interactions in the complexes involving the top selective ligands [60].

### 4.5. MD Simulations

The simulation of molecular dynamics was carried out in triplicate using the GROMACS software package (v. 2018.4) [61]. Before running the simulation, topology and parameter files were generated using ACPYPE (AnteChamber PYthon Parser interfacE) version 2022.1.3, applying the General Amber Force Field (GAFF) to the molecules of interest [62].

At the preliminary stage, the system was solvated by introducing solvent molecules into a 12-faced simulation box, ensuring a minimum spacing of 10 Å between the solvent and the box boundaries. The TIP3P water model was employed for this solvation procedure.

In the second phase, we added ions (both cations and anions) to the system to neutralize the charges, thereby simulating physiological conditions. Subsequently, we minimized the system using the steepest descent algorithm, which consisted of 50,000 steps.

In the third stage, equilibration was performed under NVT conditions (constant number of particles, volume, and temperature). A V-rescale thermostat with a time constant of 0.1 picoseconds was used for temperature control, and the particles were assigned initial velocities according to a Maxwellian distribution.

In the fourth stage, equilibration was performed under NPT conditions (constant number of particles, volume, temperature, and pressure). Thermal regulation was achieved using the V-rescale thermostat with a coupling constant of 0.1 picoseconds, while pressure was controlled through the Berendsen barostat, applying a time constant of 2.0 picoseconds. Throughout this equilibration step, the system was maintained at a target temperature of 300 K for a duration of 100 picoseconds. Following the equilibration phase, a 120-nanosecond molecular dynamics simulation was performed. Temperature control was maintained using the V-rescale thermostat with a coupling constant of 0.1 picoseconds, while pressure regulation employed the Parrinello–Rahman barostat, using a time constant of 2.0 picoseconds and maintaining a target temperature of 300 K. To assess complex stability, the root mean square deviation (RMSD), root mean square fluctuation (RMSF), and radius of gyration were computed. 

#### 4.5.1. Trypanocidal Activity In Vitro

The trypanocide was evaluated on the epimastigotes of the Mexican strains NINOA and A1 from *T. cruzi*. These strains were maintained in a liver infusion tryptose (LIT) medium supplemented with 10% fetal bovine serum (FBS) and 0.1% penicillin–streptomycin. To maintain viability, strains were transferred to a fresh medium at a concentration of 1 × 10^6^ parasites/mL once a week.

All compounds were initially prepared at a concentration of 10 mg/mL using dimethyl sulfoxide (DMSO) as a diluent. Subsequently, serial dilutions were performed with LIT medium to obtain concentrations ranging from 200 to 0.46 μM for each compound. *T. cruzi* epimastigotes (1 × 10^6^) were cultured in each well and incubated for 48 h/28 °C in a final volume of 200 μL. DMSO was included as a negative control and the reference drugs (Nfx, Bzn) as positive controls. After the incubation period, 20 μL of a resazurin solution (2.5 mM) was added to each well and incubated for 3 h. All assays were carried out in triplicate, and the half-maximal inhibitory concentration (IC_50_) value was determined by probit analysis.

#### 4.5.2. Cytotoxicity

Cytotoxicity assays were conducted on a mouse macrophage cell line (J774). The cells were cultured in RPMI medium supplemented with 10% FBS and penicillin (100 U μg/mL), streptomycin (100 U μg/mL), and glutamine (2 mM) at 37 °C in a 5% CO_2_ atmosphere, with medium replacement every 2–3 days. To assess the cytotoxicity of the compounds, 1 × 10^6^ cells were incubated with varying concentrations of the compound (ranging from 0.78 μM to 200 μM) in a final volume of 200 μL for 48 h at 37 °C in a 5% CO_2_ atmosphere. DMSO at 0.1% (the highest concentration used) served as the negative control, while control drugs were employed as positive controls. Cell metabolic activity was assessed using the MTT method, and % cell viability was calculated. The half-maximal cytotoxic concentration (CC_50_) was determined through probit analysis. Three independent assays were conducted in triplicate. The selectivity index (SI) was calculated for epimastigotes of the NINOA strain (CC_50_/IC_50_).

## 5. Conclusions

Overall, this work identified a potential inhibitor targeting the general transcription factor TBP of *T. cruzi*, potentially paving the way for developing targeted therapies for this parasitic disease. The low conservation of amino acids in this transcription factor supports the possibility of designing specific treatments that can target this essential protein while minimizing potential adverse effects on the host. The insights gained from molecular docking and molecular dynamics simulations offer a robust basis for further investigation and in vitro testing. Notably, DB00890 for *Tc*TBP emerged as a compound showing potential inhibitory effects. However, it should be noted that DB00890 displayed only moderate trypanocidal activity against *T. cruzi* epimastigotes, with a moderate selectivity index (SI values of 2.84 and 5.36) against macrophage J774.2 for the NINOA and A1 strains, respectively. These findings suggest that while DB00890 represents a promising starting point, further optimization or combination strategies will likely be required to enhance its potency and selectivity, thereby achieving clinical relevance. Therefore, future studies should focus on structural optimization and in vivo validation to better assess its therapeutic potential.

## Figures and Tables

**Table 1:** Top ten compounds with the best BFE against the TBP of *Trypanosoma cruzi*.

Compound	Target	Anti-*Trypanosoma* Effect	Binding Free Energy, Interaction Profile, and Selectivity Index (SI)	Other Functions
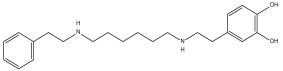 Dopexamine (DB12313)	Not Available.	Negative	−5.645 kcal/mol. **HI.** P1, A3, Y180, L183; **HB**. D177, Y180, T181. SI: −1.367	Dopexamine has been used in trials studying the diagnostic and treatment of free flap, oral cancer, hypotension, septic shock, and head and neck cancer [35].
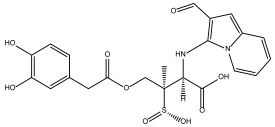 DB08116	Beta-lactamase SHV-1.	Negative	−6.465 kcal/mol. **HI.** R142, Q153; **HB.** L143, A144, V154. SI: −1.242	Beta-lactamase inhibitor [36]. Offers resistance against a variety of beta-lactam antibiotics.
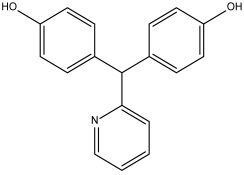 Deacetylbisacodyl (DB14232)	Not Available.	Negative	−6.422 kcal/mol. **HI.** P1, F179, Y180, L183, P184; **HB.** A4, T7. SI: −1.086	Not Available.
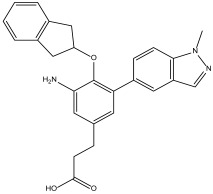 AK106-001616 (DB16058)	Not Available.	Negative	−7.416 kcal/mol. **HI.** P1, Y180, P184; **HB.** P5, T7. SI: −1.083	AK106-001616 is currently being examined in clinical trial NCT01285752 (An Investigation of AK106-001616 in Individuals Diagnosed with Rheumatoid Arthritis (RA)) [35].
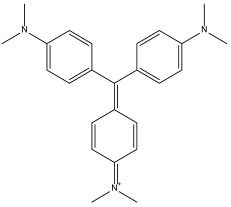 Gentian Violet (DB00406)	DNA.	Positive	−6.561 kcal/mol. **HI.** P1, Y180, L183, P184. SI: −1.046	Gentian violet at 250 µg/mL with 2 mg/mL of ascorbic acid and six hours of photoradiation (75 W) sterilized blood samples [37]. Gentian violet has been identified as an acetylcholinesterase inhibitor (AChE) [38].
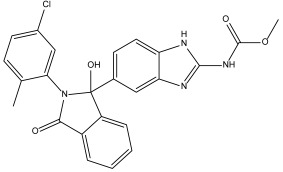 BMS-908662 (DB12854)	Not Available.	Negative	−7.741 kcal/mol. **HI.** P1, Y180, L183, P184; **HB.** P5; **Ï€-s.** Y180. SI: −1.035	BMS-908662 has been employed in clinical trials investigating its potential for treating melanoma and colorectal cancer [35].
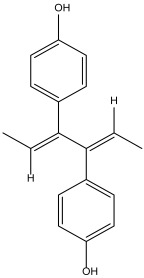 Dienestrol (DB00890)	Not Available.	Positive	−6.444 kcal/mol. **HI.** P1, Y180, L183, P184; **HB.** P1, P5, T7. SI: −1.035	Dienestrol is an estrogenic compound devoid of steroidal properties, utilized for treating atrophic vaginitis and kraurosis vulvae [35]. Some estrogens have been noted to decrease protist parasite infections, including trypanosomes [39,40].
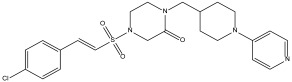 DB08745	Coagulation factor X.	Negative	−6.521 kcal/mol. **HI.** P1, Y180, L183, P184. SI: −0.985	Not available.
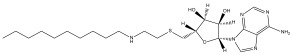 DB07413	Hydroxymycolate synthase MmaA4.	Negative	−5.476 kcal/mol. **HI.** A3, F179, Y180, L183; **HB.** N146, Y180, T181. SI: −1.035	S-Adenosylmethionine-dependent methyltransferases (AdoMet-MTs) inhibitor [41].
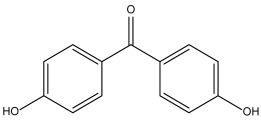 DB07635	Lanosterol 14-alpha demethylase.	Positive	−6.201 kcal/mol. **HI.** P1, Y180, L183, P184; **HB.** T7. SI: −0.977	This compound inhibits 14 alpha-demethylase (CYP51), disrupting the production of sterols and impeding the growth of *M. tuberculosis* within a mouse macrophage model [42].

**Table 2:** Trypanocide activity of compounds DB07635 and DB00890 against the epimastigotes of Mexican strains NINOA and A1 and cytotoxicity in a murine model of macrophage cells (J774.2).

ID	*T. cruzi* ^a^ IC_50_ (μM ± SD) NINOA	*T. cruzi* ^a^ IC_50_ (μM ± SD) A1	J774.2 ^b^ CC_50_ (μM ± SD)	^c^ SI NINOA	^c^ SI A1
DB07635	>200	>200	>200	ND	ND
DB00890	70.4094 ± 0.7681	37.2594 ± 0.0174	>200	2.84	5.36
Nfx	7.09 ± 0.12	19.30 ± 0.08	164.20 ± 0.25	23.15	8.50
Bzn	30.3 ± 0.03	39.08 ± 0.07	133.90 ± 0.06	4.41	3.42

^a^ IC_50_: Half-maximal inhibitory concentration. ^b^ CC_50_: Half-maximal cytotoxicity concentration. ^c^ SI: Selective index (CC_50_/IC_50_). Not determined (ND).

## Data Availability

The original contributions presented in the study are included in the article/Supplementary Material, further inquiries can be directed to the corresponding authors.

## Supplementary Materials

The following supporting information can be downloaded at: https://www.mdpi.com/article/10.3390/ph18060845/s1, Figure S1: Multiple sequence alignment of TBP orthologs highlighting the DNA-binding domain and NC2 motif; Figure S2: Root-mean-square fluctuation (RMSF) per residue of the *Tc*TBP–DB00890 complex throughout the molecular dynamics simulation; Figure S3: Comparative visualization of the *Tc*TBP binding pocket in apo and Dienestrol-bound states; Figure S4: Time-resolved map of hydrogen bond donor (HBDonor, blue) and van der Waals contact (VdWContact, yellow) interactions between Dienestrol and *Tc*TBP during the molecular dynamics simulation.

## Abbreviations

The following abbreviations are used in this manuscript:

*A. thaliana*	*Arabidopsis thaliana*
BFE	Binding free energy
Bzn	Benznidazole
CC_50_	Half-maximal cytotoxic concentration
C-terminal	Carbon terminal
DHEA	Dehydroepiandrosterone
DMSO	Dimethyl sulfoxide
DNA	Deoxyribonucleic acid
*E. cuniculi*	*Encephalitozoon cuniculi*
*E. histolytica*	*Entamoeba histolytica*
FBS	Fetal bovine serum
FDA	Food and Drug Administration
*G. lamblia*	*Giardia lamblia*
GAFF	General Amber Force Field
*Gl*TBP	*Giardia lamblia* TATA Binding Protein
*Hs*TBP	*Homo sapiens* TATA Binding Protein
IC_50_	Half-maximal inhibitory concentration
kcal/mol	Kilocalories per mole
*L. mexicana*	*Leishmania mexicana*
LIT medium	Liver infusion tryptose medium
mRNA	Messenger RNA
µM	Micromolar
MTT	3-(4,5-dimethylthiazol-2-yl)-2,5-diphenyltetrazolium bromide
NC2	Negative cofactor 2
NCBI	National Center for Biotechnology Information
Nfx	Nifurtimox
ns	Nanoseconds
NTD	Neglected Tropical Disease
*P. falciparum*	*Plasmodium falciparum*
PDBe	Protein Data Bank Europe
PLIP	Protein–Ligand Interaction Profiler
RCSB PDB	Research Collaboratory for Structural Bioinformatics Protein Data Bank
RMSD	Root mean square deviation
RMSF	Root mean square fluctuation
RNA	Ribonucleic acid
RPMI medium	Roswell Park Memorial Institute medium
rRNA	Ribosomal RNA
*S. cerevisae*	*Saccharomyces cerevisae*
SI	Selective index
snRNA	Small nuclear RNA
*T. cruzi*	*Trypanosoma cruzi*
TBP	TATA Binding Protein
*Tc*TBP	*Trypanosoma cruzi* TATA Binding Protein
TFIIA	Transcription factor IIA
TFIIB	Transcription factor IIB
tRNA	Transcription RNA
UCSF Chimera	University of California San Francisco Chimera
W	Watts
WHO	World Health Organization
