# Supplementary material for: In Silico Investigation of TATA-Binding Protein as a Therapeutic Target for Chagas Disease: Insights into FDA Drug Repositioning
Source: Pharmaceuticals (Basel). 2025 Jun 4;18(6):845. doi: 10.3390/ph18060845 (PMC12195786; doi:10.3390/ph18060845)
Supplement: Supplementary file 1 [file pharmaceuticals-18-00845-s001.zip › pharmaceuticals-3627097-supplementary.pdf]

Supplementary Material

```
CLUSTAL O(1.2.4) multiple sequence alignment
Giardia_intestinalis                   ------------------------------------------------------------         0
Entamoeba_histolytica_XP_654935        ------------------------------------------------------------         0
Encephalitozoon_cuniculi_3OCI_1        ------------------------------------------------------------         0
Drosophila_melanogaster_NP_523805      MDQMLSPNFSIPSIG---TPLHQMEADQQIVANPVYHPPAVSQPDSLMPAPGSSSVQHQQ         57
Homo_sapiens_1JFI                      ------------------------------------------------------------         0
Takifugu_flavidus_XP_056871520         ----MDQNNSIPGFQGLASPQGAMTP-----SMPIFSP--------MMP-----------         32
ARABIDOPSIS_THALIANA_1QNA              ------------------------------------------------------------         0
Saccharomyces_cerevisiae_1NH2          ------------------------------------------------------------         0
Trypanosoma_cruzi                      -----------------------MDDD---------------------------------         4
Leishmania_mexicana                    -----------------------MDDD---------------------------------         4


Giardia_intestinalis                   ------------------------------------------------------------         0
Entamoeba_histolytica_XP_654935        ------------------------------------------------------------         0
Encephalitozoon_cuniculi_3OCI_1        ------------------------------------------------------------         0
Drosophila_melanogaster_NP_523805      QQQQSDASGGSGLFGHEPSLPLAH-KQMQSYQPSASYQQQQQQQLQSQAPGGGGSTPQS         116
Homo_sapiens_1JFI                      ------------------------------------------------------------         0
Takifugu_flavidus_XP_056871520         --------YGSGLTPQ----PVQNTNSLSILEEQQRQQQQQQAQQANTGIPGTSGTTPQL         80
ARABIDOPSIS_THALIANA_1QNA              ------------------------------------------------------------         0
Saccharomyces_cerevisiae_1NH2          ------------------------------------------------------------         0
Trypanosoma_cruzi                      ----F-DNFGGD-FDLDENEFLEDAAN----DPS-------DA-AEFDY-----------         35
Leishmania_mexicana                    ----Y-AFFESD-NLVEEELPVIGAFP----DP---------V-AEFGGVVGTTGAIGDL         44


Giardia_intestinalis                   ---------------------------------------------------------AVQ         3
Entamoeba_histolytica_XP_654935        MSSQSSPIS----------------------LTNNDLEISSN---NNKPIIKQLPVPTNE         35
Encephalitozoon_cuniculi_3OCI_1        -------------MGSSHHHHHHSSGLV---PRGSHMDAPD-----ISYEHQETSVPNRS         39
Drosophila_melanogaster_NP_523805      MMQPQTPQSMMAHMMPMSERSVGGSGAG---GGGDALSNIHQTMGPSTPMTPATPGSADP         173
Homo_sapiens_1JFI                      -------------------------------------------------------GSHMS         5
Takifugu_flavidus_XP_056871520         FHSQA----------------IAGTTTT---ALPGNTPLYNTPLTPMTPITPATPASESS         121
ARABIDOPSIS_THALIANA_1QNA              --------------------------------------MTDQGL---EGSNPVDLSKHPS         19
Saccharomyces_cerevisiae_1NH2          -----------------------------------------------------------S         1
Trypanosoma_cruzi                      --------------TLFSSAH----NVSRPNDDNDNTN-SEEVRAATPPNIQELFPNAAP         76
Leishmania_mexicana                    --------------ALTADGHLAAAAATLPGGDGAAPKLDFDELPPPIKNVQEFLPNVHP         90


Giardia_intestinalis                   GLSVKVVGYNCRFSLGFNVDMRLLAASLLTADYNPR--YPTVRVRLTSPQCCISVSYHGH         61
Entamoeba_histolytica_XP_654935        IEKPVIQNIVATVELDCTINLQDVVRRVRNAEYNPK-RFGALIIRITNPKTTALVFHSGK         94
Encephalitozoon_cuniculi_3OCI_1        GIIPTLQNVVATVNLSCKLDLKNIALRARNAEYNPK-RFAAVIMRIREPKTTALIFASGK         98
Drosophila_melanogaster_NP_523805      GIVPQLQNIVSTVNLCCKLDLKKIALHARNAEYNPK-RFAAVIMRIREPRTTALIFSSGK         232
Homo_sapiens_1JFI                      GIVPQLQNIVSTVNLGCKLDLKTIALRARNAEYNPK-RFAAVIMRIREPRTTALIFSSGK         64
Takifugu_flavidus_XP_056871520         GIVPQLQNIVSTVNLGCKLDLKTIALRARNAEYNPK-RFAAVIMRIREPRTTALIFSSGK         180
ARABIDOPSIS_THALIANA_1QNA              GIVPTLQNIVSTVNLDCKLDLKAIALQARNAEYNPK-RFAAVIMRIREPKTTALIFASGK         78
Saccharomyces_cerevisiae_1NH2          GIVPTLQNIVATVTLGCRLDLKTVALHARNAEYNPK-RFAAVIMRIREPKTTALIFASGK         60
Trypanosoma_cruzi                      DTMPVIVGIIAQAKLGVGVDLCALSCATRNVEFVPRNRTPAATMRLHEPTAVVLVRTSGF         136
Leishmania_mexicana                    DAFPVVVAVQAQASIPVGINLAELSCATRNVEYMPNNRIPSATMRLHEPTAVVMMHNSGA         150
                                            :    .   :   :::  :     ..:: *.    :  :*: .*     :   *

Giardia_intestinalis                   CTIFGCESVAQAATAAAVFLKLLNEIEEFVGLARPSPLTVVSITCLTDLGHGIRLDAAAA         121
Entamoeba_histolytica_XP_654935        LVVTGGKTVDDSRLAGRKYARILQR-LGYN--VKFNHFKIQNVVASCDMKFAISLKELIQ         151
Encephalitozoon_cuniculi_3OCI_1        MVITGAKSEKSSRMAAQRYAKIIHK-LGFN--ATFDDFKIQNIVSSCDIKFSIRLEGLAY         155
Drosophila_melanogaster_NP_523805      MVCTGAKSEDDSRLAARKYARIIQK-LGFP--AKFLDFKIQNMVGSCDVKFPIRLEGLVL         289
Homo_sapiens_1JFI                      MVCTGAKSEEQSRLAARKYARVVQK-LGFP--AKFLDFKIQNMVGSCDVKFPIRLEGLVL         121
Takifugu_flavidus_XP_056871520         MVCTGAKSEEQSRLAARKYARVVQK-LGFP--AKFLDFKIQNMVGSCDVRFPIRLEGLVL         237
ARABIDOPSIS_THALIANA_1QNA              MVCTGAKSEDFSKMAARKYARIVQK-LGFP--AKFKDFKIQNIVGSCDVKFPIRLEGLAY         135
Saccharomyces_cerevisiae_1NH2          MVVTGAKSEDDSKLASRKYARIIQK-IGFA--AKFTDFKIQNIVGSCDVKFPIRLEGLAF         117
Trypanosoma_cruzi                      MSIIGAASVGEAKQATELAARIIRKALSLD--ITTVQFRVRSVTARFDVGHPIRLEELAQ         194
Leishmania_mexicana                    LSIIGAASVSEARQAAELAARIIRKALNLN--FSSLKFRVRSIAARFNVCSPIRLDKLAA         208
                                           *  :   :  *     :::..            : : .:.   ::   * *.

Giardia_intestinalis                   ATIS-------IFSSAMYQPEIMPSLQVVFKIAERN--------ICCSVFANGQVTIVGA         166
Entamoeba_histolytica_XP_654935        L----------APKITKYEPEIFPGVV--YRLADPK--------MVLLIFASGKIVFTGG         191
Encephalitozoon_cuniculi_3OCI_1        A----------HSNYCSYEPELFPGLI--YRMVKPK--------IVLLIFVSGKIVLTGA         195
Drosophila_melanogaster_NP_523805      T----------HCNFSSYEPELFPGLI--YRMVRPR--------IVLLIFVSGKVVLTGA         329
Homo_sapiens_1JFI                      T----------HQQFSSYEPELFPGLI--YRMIKPR--------IVLLIFVSGKVVLTGA         161
Takifugu_flavidus_XP_056871520         T----------HQQFSSYEPELFPGLI--YRMIKPR--------IVLLIFVSGKVVLTGA         277
ARABIDOPSIS_THALIANA_1QNA              S----------HAAFSSYEPELFPGLI--YRMKVPK--------IVLLIFVSGKIVITGA         175
Saccharomyces_cerevisiae_1NH2          S----------HGTFSSYEPELFPGLI--YRMVKPK--------IVLLIFVSGKIVLTGA         157
Trypanosoma_cruzi                      H----------EGIFCSYEPDRFSGCI--VRLAGNS--HDNQWQVSCTVFVTGKVSILGA         240
Leishmania_mexicana                    YKLDPAMSIGVAKLQVSYEPERFNGCV--LRLVGKSSRGDNQWSVSCSVFVTGKVQLMGA         266
                                                        *:*: : .     ::            :   :*..*:: : *.

Giardia_intestinalis                   RNIFDARDVITKLYEGLFDYFIT----   189
Entamoeba_histolytica_XP_654935        KEIEQINKAFSEIYKILLQVANNDN--   216
Encephalitozoon_cuniculi_3OCI_1        KVRDDIYQAFNNIYPVLIQHRKA----   218
Drosophila_melanogaster_NP_523805      KVRQEIYDAFDKIFPILKKFKKQS---   353
Homo_sapiens_1JFI                      KVRAEIYEAFENIYPILKGFRKTT---   185
Takifugu_flavidus_XP_056871520         KVRAEIYEAFENIYPILKGFRKTT---   301
ARABIDOPSIS_THALIANA_1QNA              KMRDETYKAFENIYPVLSEFRKIQQ--   200
Saccharomyces_cerevisiae_1NH2          KQREEIYQAFEAIYPVLSEFRKM----   180
Trypanosoma_cruzi                      RSQEELQDAFYTLLPILAQYAKK----   263
Leishmania_mexicana                    RSMDELRFAFNAFVPIIAKNLDERKTA   293
                                       :   :   .:  :   :
```

Figure 1. Multiple sequence alignment of TBP orthologs highlighting the DNA-binding domain and NC2 motif. The alignment was performed using the Clustal Omega algorithm, guided by an initial structural alignment. Based on this, the DNA-binding (C-terminal) domains were defined for proteins lacking experimentally resolved structures. Amino acid residues corresponding to the NC2 interaction motif are highlighted in yellow.

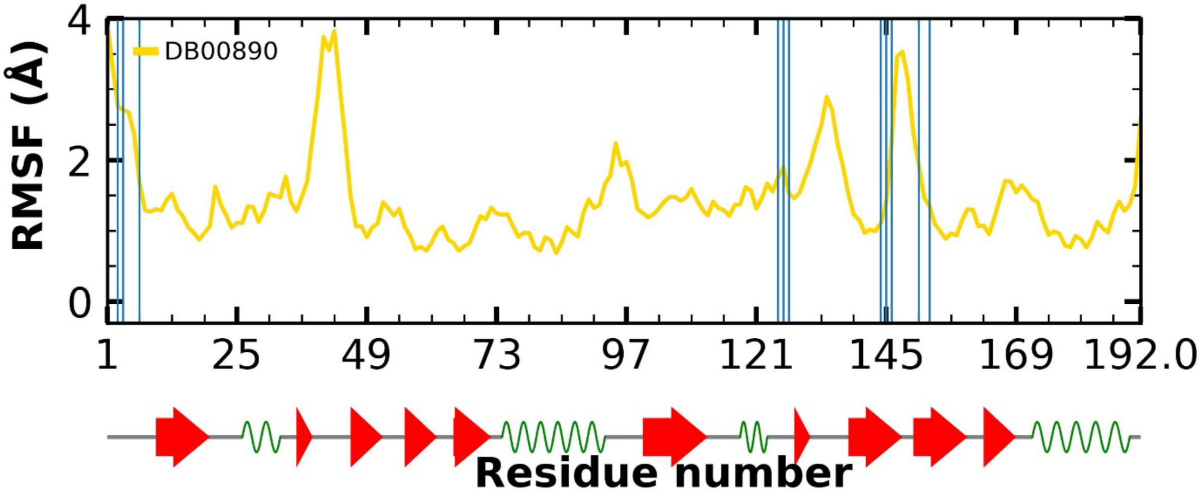

Figure 2. Root-mean-square fluctuation (RMSF) per residue of the TcTBP–DB00890 complex throughout the molecular dynamics simulation.

The yellow line represents atomic fluctuations of the Cα atoms over time. Vertical blue lines highlight the key amino acid residues identified in the analysis as interacting with compound **DB00890**. The secondary structure of *Tc*TBP is shown below, with red arrows representing β-sheets, green sinusoidal lines indicating α-helices, and grey lines unstructured regions to contextualize flexibility and binding sites. This modified figure focuses on **DB00890**, the compound with the most favorable *in silico* performance.

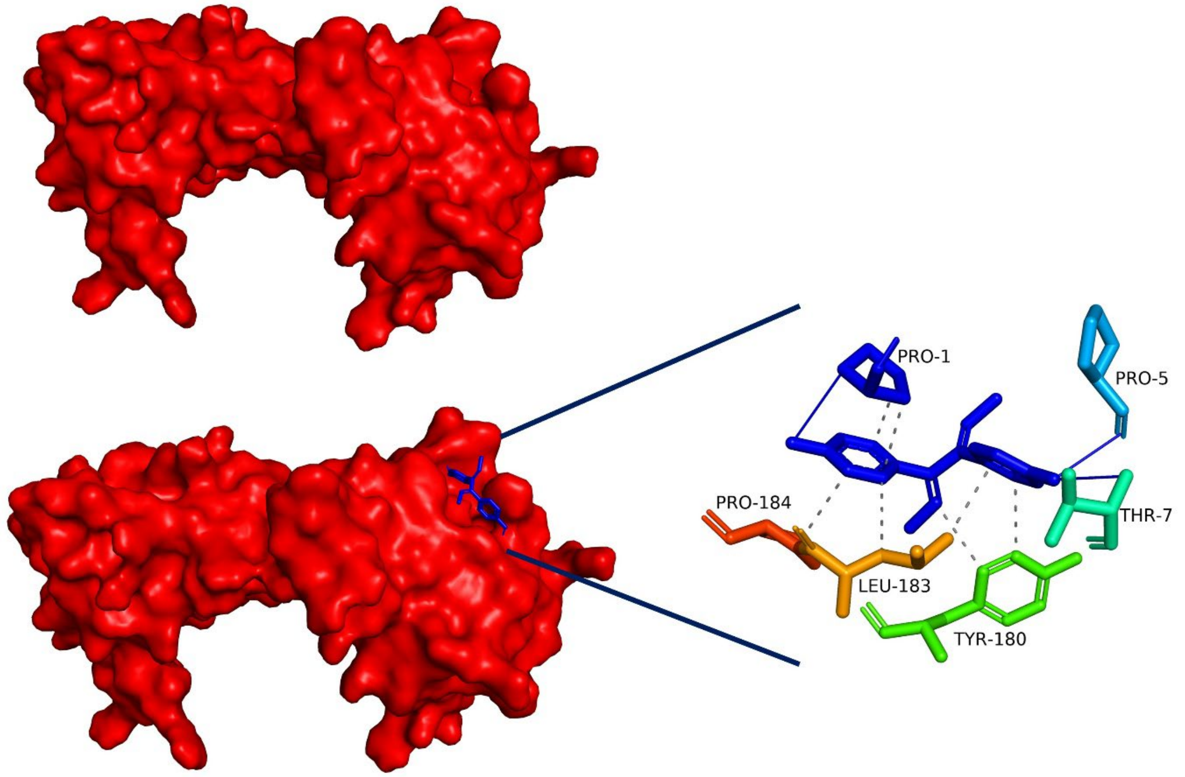

Figure 3. Comparative visualization of the *Tc*TBP binding pocket in apo and Dienestrol-bound states.

***Left panel*:** Surface representation of *Tc*TBP showing the binding site without the ligand (top) and with **Dienestrol** (**DB00890**) bound (bottom), highlighting pocket occupancy upon ligand binding. *Right panel*: Close-up view of the binding site showing **Dienestrol** (blue sticks) interacting with key *Tc*TBP residues. Pro-1, Tyr-180, Leu-183, and Pro-184 establish hydrophobic interactions, while Pro-1, Pro-5, and Thr-7 participate in hydrogen bonding. Hydrophobic interactions are represented by dashed lines, and hydrogen bonds by solid lines. **Dienestrol** exhibited a binding free energy of -6.376 ± 0.067 kcal/mol and a selectivity value of -0.967 ± 0.067 relative to *Hs*TBP. Interestingly, synthetic non-steroidal estrogens, such as Dienestrol, have been reported to reduce the severity of infections caused by protozoan parasites, including trypanosomes. Similarly, administration of dehydroepiandrosterone (DHEA) has been associated with a reduction in bloodstream trypomastigote load.

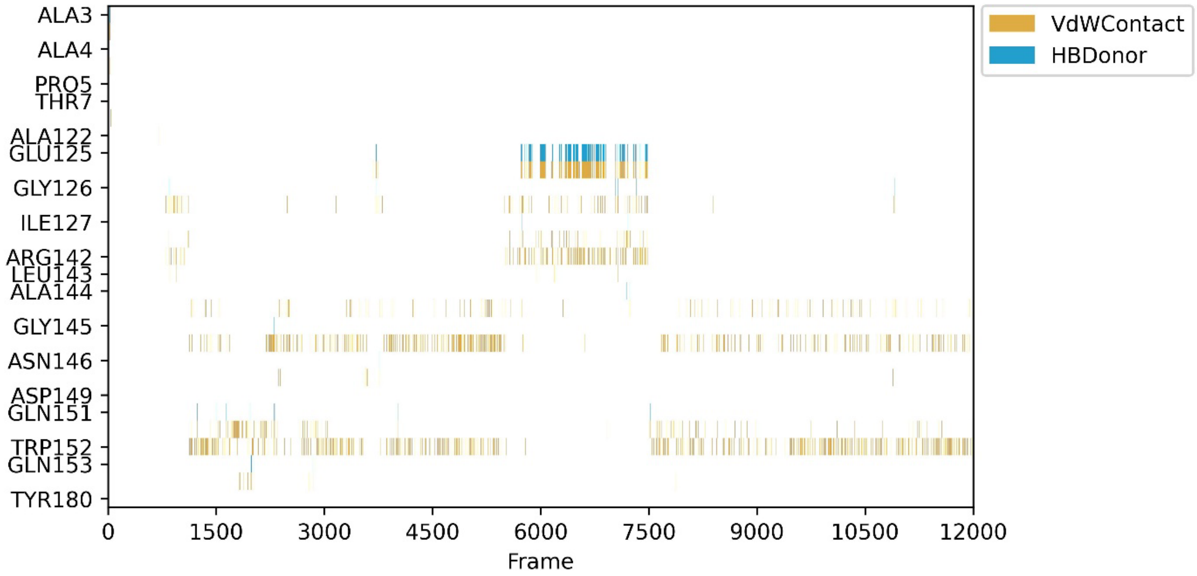

Figure 4. Time-resolved map of hydrogen bond donor (HBDonor, blue) and van der Waals contact (VdWContact, yellow) interactions between Dienestrol and *Tc*TBP during the molecular dynamics simulation.

The plot illustrates the persistence and distribution of interactions over 12,000 simulation frames. Persistent van der Waals contacts are observed with residues Gly145, Gln151, and Trp152, while transient hydrogen bonding events are detected with Glu125. Notably, despite being identified in the static docking model, interactions with Pro1, Pro5, and Thr7 are not sustained over time in the simulation. This highlights the dynamic nature of ligand binding and underscores the relevance of combining static and dynamic analyses to fully characterize interaction profiles.
